# Bioinformatic analysis reveals an association between Metadherin with breast cancer prognosis and tumor immune infiltration

**DOI:** 10.1038/s41598-024-52403-x

**Published:** 2024-01-23

**Authors:** Lixian Yang, Liu Yang, Fanting Kong, Shiyu Zhang, Pengpeng Pu, Xiaowei Li, Zhenchuan Song

**Affiliations:** 1 Breast Center, The Fourth Hospital of Hebei Medical University, 169 Changjiang Avenue, Shijiazhuang, 050000 Hebei People’s Republic of China; 2 Department of Breast Surgery, Xingtai People’s Hospital, No. 818 Xiangdu district, Xingtai, 054000 Hebei People’s Republic of China

**Keywords:** Cancer, Computational biology and bioinformatics

## Abstract

Breast cancer metastasis and invasion are both promoted by the oncoprotein Metadherin (MTDH). However, the the role of Metadherin in breast cancer progression and its role in the immune microenvironment. Are not clear. A bioinformatic analysis was performed to demonstrate the prognostic value of Metadherin in BC. In the present study, we found that Metadherin is overexpressed in BC and is significantly correlated with individual cancer stage, age, subclasses, menopause and nodal metastasis status. Metadherin overexpression was associated with a significant decrease in OS and DSS. Cox multivariate analysis indicated that Metadherin was an independent negative prognostic indicator for OS and DSS. Moreover, Metadherin hypomethylation status was associated with poor prognosis. A negative correlation was also noted between Metadherin overexpression and the number of plasmacytoid dendritic cells, cluster of differentiation 8^+^ T cells, and natural killer cells. Association patterns varied with different subtypes. Various associations between Metadherin levels and immune cell surface markers were revealed. A total of 40 groups of BC and adjacent normal breast tissue samples were collected. Metadherin mRNA was detected by PCR, and its expression levels in BC tissues were significantly increased compared with those noted in normal tissues. The expression levels of Metadherin were also measured in normal and BC cell lines, respectively, and similar conclusions were obtained. The Metadherin mRNA levels were knocked down in SK-BR3 and MDA-MB-231 cell lines and the cell proliferative and migratory activities were determined using Cell Counting Kit-8 and scratch assays, respectively. The results indicated that the cell proliferative and migratory abilities were reduced following knockdown of Metadherin expression. Therefore, Metadherin may be considered as a novel prognostic biomarker in BC.

## Introduction

Breast cancer (BC) is a major threat to women’s health and is one of the most common types of cancer worldwide^1^. Although BC diagnosis and treatment have advanced dramatically, various patients relapse to this disease or their corresponding tumors develop resistance against chemotherapy^2^. Therefore, improvements in diagnostic and therapeutic methods are urgently required^3^. In patients with BC, tumor stage, nodal and metastatic system (TNM), and molecular classification play a significant role in treatment decisions and prognosis^4^. Despite similar treatment regimens, similar cancer stages and molecular subtypes may result in different clinical outcomes^5^. As a result, the current staging system cannot adequately predict prognosis for patients with BC due to their biological heterogeneity. The clinical outcome of the patient can also be influenced by the characteristics of the tumor cells. Prognostic markers based on these features may support the current TNM staging system, result in more accurate clinical prediction, and allow improved treatment planning^5^.

Metadherin (MTDH) is also known as astrocyte elevated gene (AEG1)^6^. Tumor cells have been shown to express Metadherin at high levels and to be closely related to cell proliferation, apoptosis, and migration in breast cancer, nasopharyngeal carcinoma, colorectal cancer and ovarian cancer^7–10^. Total knockout of Metadherin expression in mice does not affect embryogenesis or postnatal development; however, it severely affects mammary tumor formation^11^. Metadherin/AEG1 whole-body knockdown studies in prostate, liver, lung, and colorectal cancers yielded similar results^12–14^. These findings demonstrate that Metadherin is specifically required for the development of malignancy, whereas it is dispensable for normal development or homeostasis, underscoring the rational of its targeting for cancer therapy. However, the biochemical and molecular mechanisms of Metadherin, and the relationship between Metadherin expression and tumor immune cell infiltration in BC are still not very clear.

By using bioinformatics, the present study intended to assess and investigate Metadherin expression in patients with BC and to explore its association with clinicopathology and prognosis, its underlying molecular mechanism and its relationship with immune cell infiltration.

## Results

### Aberrant upregulation of Metadherin expression in BC

**Figure 1:**
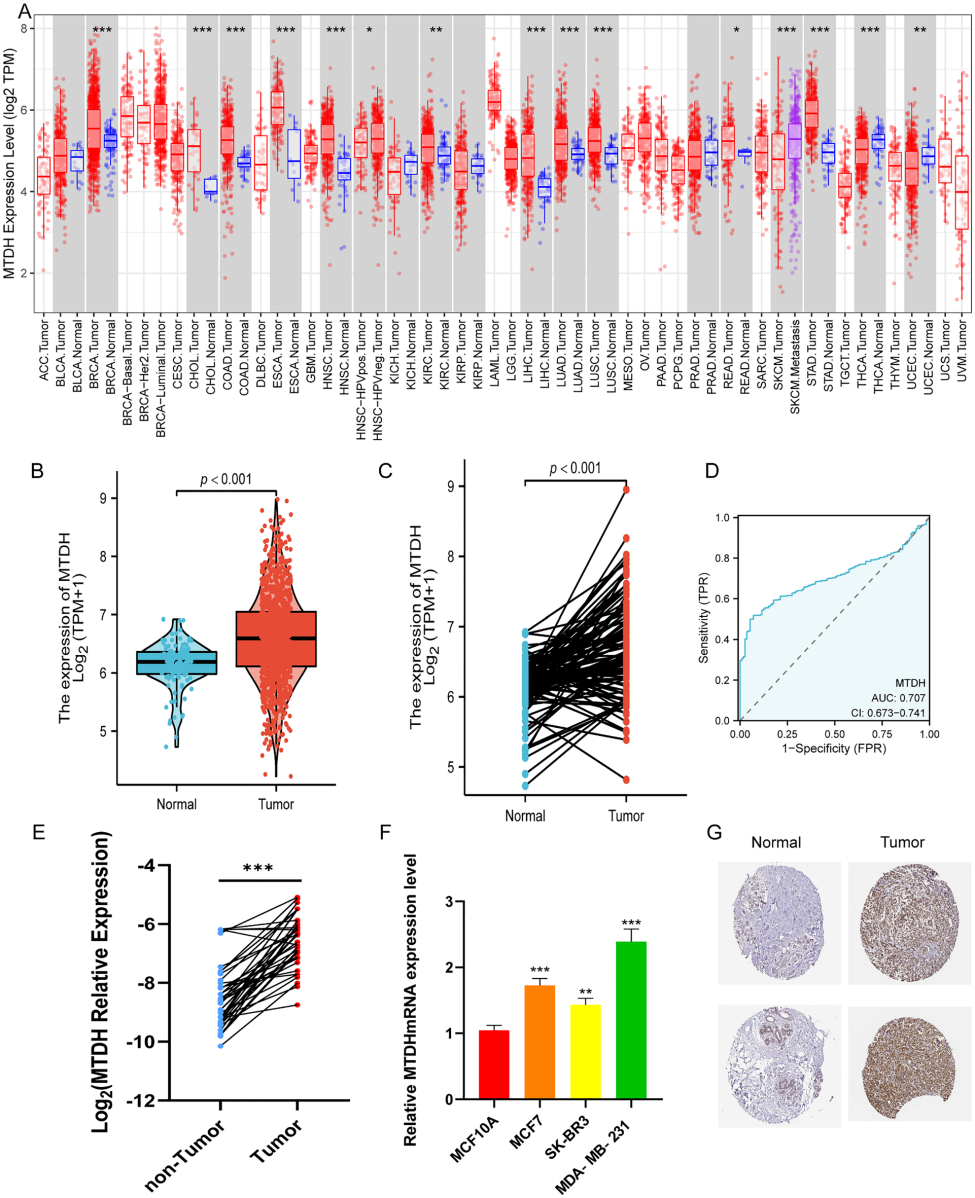
Expression levels of Metadherin in BRCA. (**A**) Expression levels of Metadherin (mean ± SD) in 33 types of cancer based on bioinformatic data. (**B**) Unpaired analysis of Metadherin expression (mean ± SD) between tumor and normal tissues in 1087 patients with BRCA. (**C**) Paired analysis of Metadherin expression (scatter points represent expression levels of individual samples) between tumor and normal tissues (n = 113). (**D**) A ROC curve was used to establish the efficiency of Metadherin mRNA expression levels on distinguishing BRCA tumor from non-tumor tissues. The X-axis represents the false positive rate and the Y-axis represents the true positive rate. (**E**) Comparison of Metadherin mRNA expression levels between normal and tumor tissues. (**F**) Relative expression levels of Metadherin mRNA in BC cells and a normal breast cell line. (**G**) Metadherin protein expression levels in samples of breast cancer tissues and normal breast tissue (HPA). Unpaired/paired Student’s *t* test was used to analyze the data. *P < 0.05, **P < 0.01, ***P < 0.001 vs. normal. *BRCA* breast adenocarcinoma, *TCGA* the cancer genome atlas, *ROC* receiver operating characteristic, *BC* breast cancer, *ns* not significant, *HPA* human protein atlas.

The analysis of the TIMER database included analysis of Metadherin expression profiles among various cancer types. The analysis indicated that the expression levels of Metadherin were significantly upregulated in the majority of cancer tissues, including BRCA, cholangiocarcinoma, colon adenocarcinoma, esophageal carcinoma, liver hepatocellular carcinoma, lung adenocarcinoma, lung squamous cell carcinoma, and stomach adenocarcinoma compared with those noted in their corresponding normal tissues (Fig. 1A). A total of 1087 BC and 113 normal breast tissues were collected from the TCGA‐BRCA datasets to examine the expression levels of Metadherin in BC and normal tissues in more detail. A significant difference was found between normal and BC tissues with regard to the expression levels of Metadherin (P < 0.001; Fig. 1B). The comparison of 113 BC tissues with their matched adjacent normal tissues demonstrated that BC tissues expressed significantly higher levels of Metadherin than those of normal tissues (P < 0.001; Fig. 1C). Furthermore, ROC curves were used to evaluate the effectiveness of the AUC corresponding to the Metadherin mRNA expression levels in detecting BC tissues. With an AUC of 0.707, Metadherin could serve as a biomarker for distinguishing BC from non-tumor tissues (Fig. 1D). RT-qPCR analysis revealed that forty BC tissues expressed considerably higher levels of Metadherin than those of normal breast tissues (P < 0.001; Fig. 1E). In addition, RT-qPCR analysis showed that all three types of BC cells as MCF7, SK-BR3 and MDA-MB-231 cells, tested expressed significantly higher levels of Metadherin than those noted in MCF10A (Fig. 1F), which was consistent with the aforementioned results. Based on the HPA database, IHC was used to verify Metadherin protein expression in normal breast and tumor tissues (Fig. 1G).

### Association between Metadherin expression and clinicopathological variables

**Figure 2:**
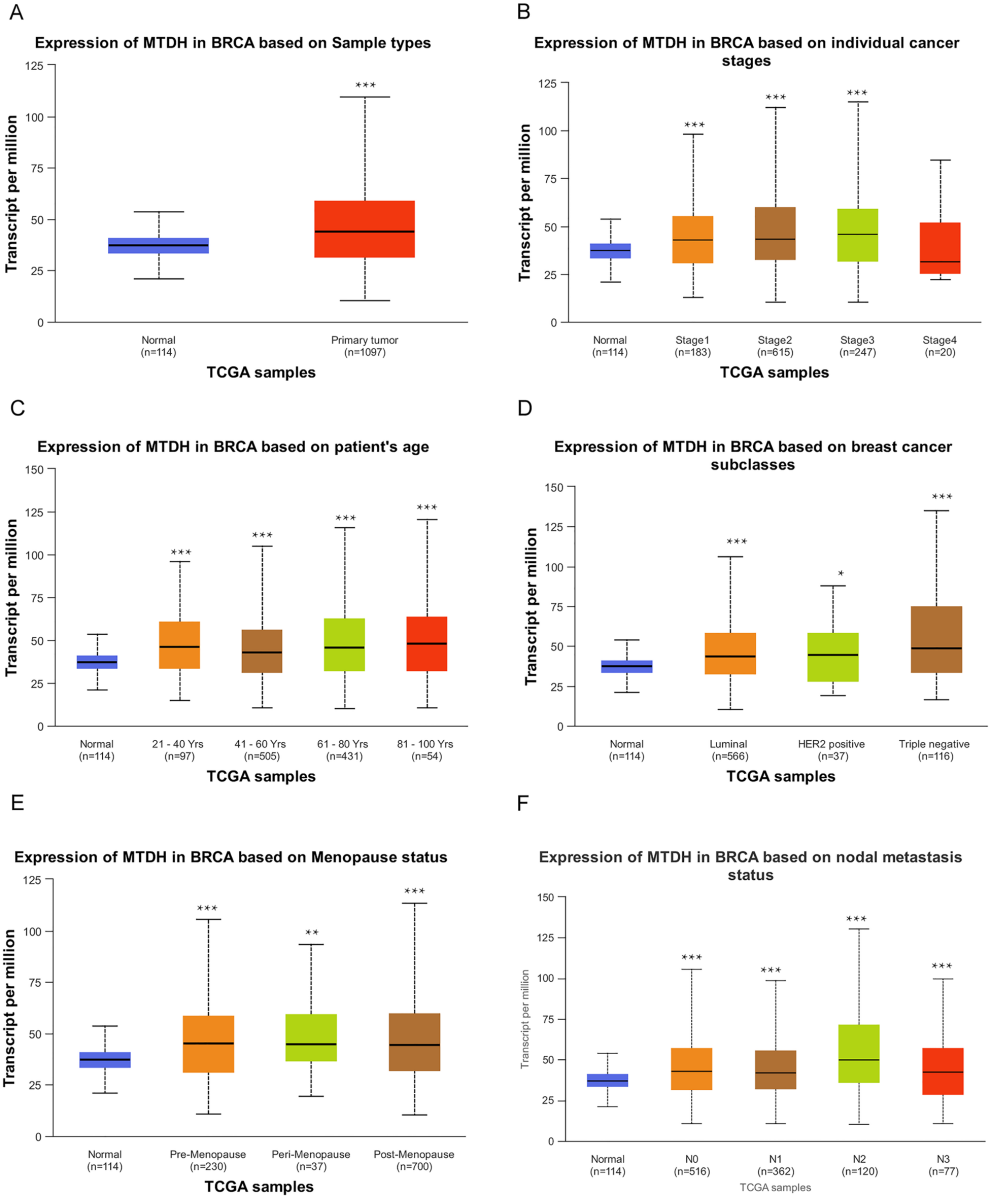
Associations between Metadherin expression and clinicopathological characteristics. The data are shown for (**A**) sample types; (**B**) individual cancer stages; (**C**) age; (**D**) breast cancer subclasses; (**E**) menopause status; (**F**) nodal metastasis status. Unpaired Student’s *t* test was used to analyze the data. HER2, human epidermal growth factor receptor 2. UALCAN, University of Alabama at Birmingham Cancer.

According to Table 1 and Fig. 2, Metadherin expression was significantly associated with individual cancer stages, age, subclasses, menopause status and nodal metastasis status. Univariate logistic regression analysis indicated certain clinicopathological differences between the groups with high and low Metadherin expression, including histological type [Odds ratio (OR) = 0.237, 95% confidence interval (CI)   0.166–0.334, P < 0.001], PAM50 (OR = 1.702, 95% CI 1.289–2.256, P < 0.001), estrogen receptor (ER) status (OR = 0.625, 95% CI 0.466–0.837, P = 0.002), and progesterone receptor (PR) status (OR = 0.679, 95% CI 0.523–0.881, P = 0.004; Table 2).

**Table 1:** Clinicopathological characteristics of high- and low- Metadherin expression groups.

Characteristics	Low expression of MTDH	High expression of MTDH	P value
n	543	544	
Pathologic T stage, n (%)			0.135
T1	136 (12.5%)	142 (13.1%)	
T2	306 (28.2%)	325 (30%)	
T3	83 (7.7%)	57 (5.3%)	
T4	18 (1.7%)	17 (1.6%)	
Pathologic N stage, n (%)			0.054
N0	263 (24.6%)	253 (23.7%)	
N1	187 (17.5%)	172 (16.1%)	
N2	45 (4.2%)	71 (6.6%)	
N3	43 (4%)	34 (3.2%)	
Pathologic M stage, n (%)			0.119
M0	429 (46.4%)	476 (51.5%)	
M1	13 (1.4%)	7 (0.8%)	
Pathologic stage, n (%)			0.432
Stage I	95 (8.9%)	87 (8.2%)	
Stage II	315 (29.6%)	304 (28.6%)	
Stage III	117 (11%)	127 (11.9%)	
Stage IV	12 (1.1%)	6 (0.6%)	
Race, n (%)			0.138
Asian	33 (3.3%)	27 (2.7%)	
Black or African American	105 (10.5%)	77 (7.7%)	
White	376 (37.7%)	379 (38%)	
Age, n (%)			0.978
≤ 60	301 (27.7%)	302 (27.8%)	
> 60	242 (22.3%)	242 (22.3%)	
Histological type, n (%)			< 0.001
Infiltrating ductal carcinoma	331 (30.9%)	445 (41.6%)	
Infiltrating lobular carcinoma	149 (13.9%)	56 (5.2%)	
Mixed histology (please specify)	13 (1.2%)	16 (1.5%)	
Mucinous carcinoma	13 (1.2%)	4 (0.4%)	
Other, specify	27 (2.5%)	16 (1.5%)	
PR status, n (%)			0.354
Negative	163 (15.8%)	179 (17.3%)	
Positive	351 (33.9%)	341 (33%)	
ER status, n (%)			0.425
Negative	114 (11%)	126 (12.2%)	
Positive	402 (38.8%)	395 (38.1%)	
HER2 status, n (%)			0.708
Negative	277 (38.6%)	283 (39.5%)	
Positive	75 (10.5%)	82 (11.4%)	
Menopause status, n (%)			0.314
Pre	104 (10.7%)	126 (12.9%)	
Peri	20 (2%)	20 (2%)	
Post	360 (36.9%)	346 (35.5%)	
OS event, n (%)			< 0.001
Alive	488 (44.9%)	447 (41.1%)	
Dead	55 (5.1%)	97 (8.9%)	
DSS event, n (%)			0.025
No	506 (47.4%)	476 (44.6%)	
Yes	33 (3.1%)	52 (4.9%)	
PFI event, n (%)			0.135
No	478 (44%)	462 (42.5%)	
Yes	65 (6%)	82 (7.5%)	

The Wilcoxon rank-sum test was used to analyze the data.

**Table 2:** Associations of Metadherin expression with clinicopathological characteristics of patients (n = 1087).

Characteristics	Total (N)	Odds ratio (OR)	P value
T stage (T2 & T3 & T4 vs. T1)	1080	1.065 (0.810–1.400)	0.651
N stage (N1 & N2 & N3 vs. N0)	1064	1.001 (0.787–1.273)	0.993
M stage (M1 vs. M0)	922	0.389 (0.137–0.978)	0.055
Pathologic stage (stage III & stage IV vs. stage I & stage II)	1060	1.213 (0.917–1.607)	0.177
Histological type (infiltrating lobular carcinoma vs. infiltrating ductal carcinoma)	977	0.237 (0.166–0.334)	< 0.001
PR status (positive vs. negative)	1030	0.679 (0.523–0.881)	0.004
ER status (positive vs. negative)	1033	0.625 (0.466–0.837)	0.002
HER2 status (indeterminate vs. negative)	570	1.370 (0.432–4.678)	0.594
Menopause status (peri & post vs. pre)	972	1.009 (0.750–1.357)	0.954
radiation_therapy (yes vs. no)	987	1.209 (0.940–1.555)	0.140
PAM50 (Her2 & basal vs. LumA & LumB)	1043	1.702 (1.289–2.256)	< 0.001

Logistic regression were used to analyze the data.

### Expression of Metadherin and methylation

**Figure 3:**
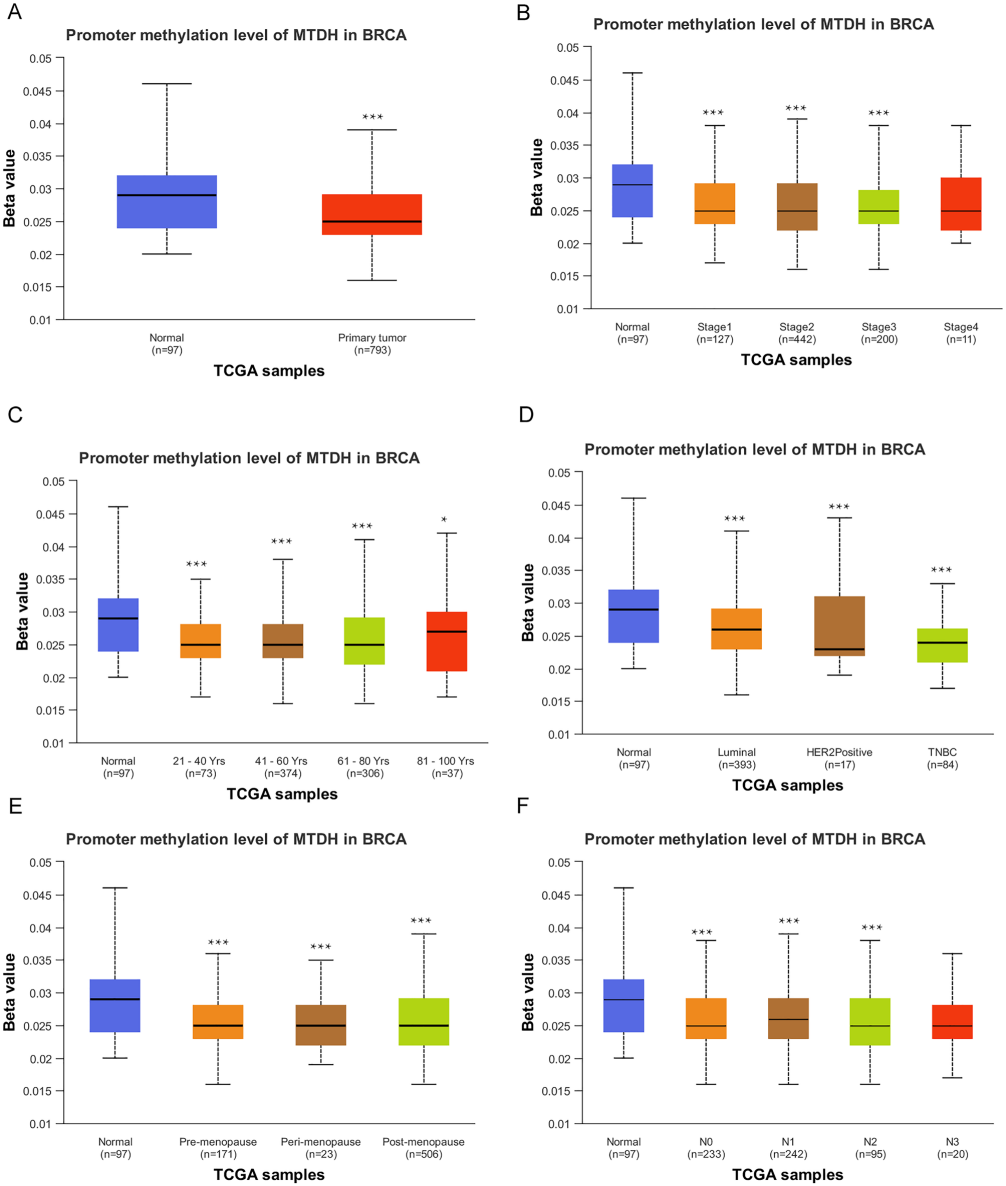
DNA methylation levels of Metadherin and its effect on the prognosis of patients with BC. The data are shown for (**A**) sample types; (**B**) individual cancer stages; (**C**) age; (**D**) breast cancer subclasses; (**E**) menopause status; (**F**) nodal metastasis status. Unpaired Student’s *t* test was used to analyze the data. BC, breast cancer; UALCAN, University of Alabama at Birmingham Cancer.

Online tools were also used to investigate the correlation between Metadherin expression levels and the methylation status noted in BC tissues. According to the UALCAN database, BC tumor tissues exhibited significantly lower DNA methylation levels than normal breast tissues (P < 0.001; Fig. 3A). DNA methylation levels of Metadherin was significantly associated with individual cancer stages (P < 0.001; Fig. 3B), age (P < 0.001; Fig. 3C), subclasses (P < 0.001; Fig. 3D), menopause status (P < 0.001; Fig. 3E) and nodal metastasis status (P < 0.001; Fig. 3F).

### Prognostic value of Metadherin expression in BC

**Figure 4:**
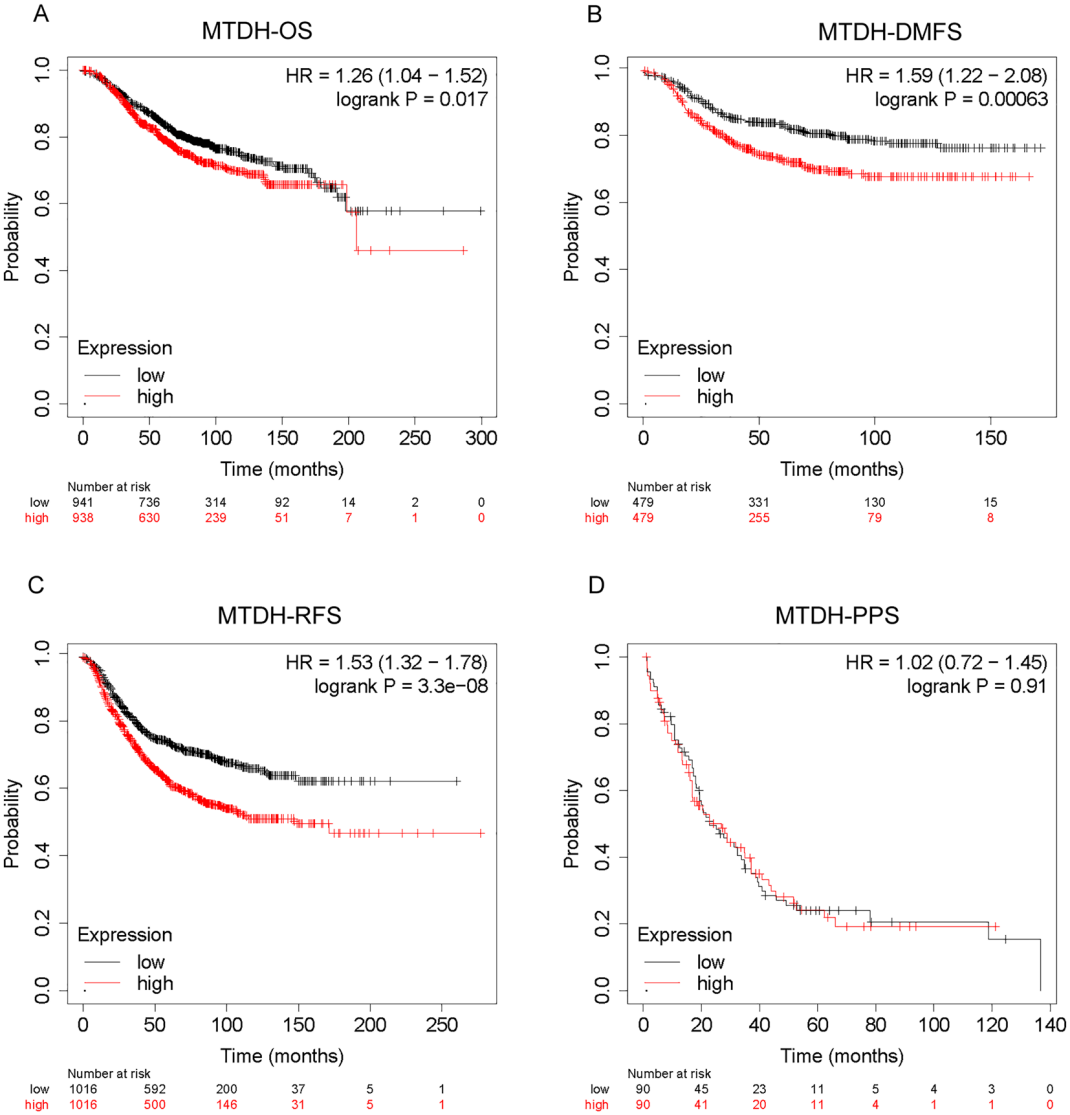
The prognostic values of Metadherin expression in patients with BC were evaluated by the Kaplan–Meier method. OS (**A**), DMFS (**B**), RFS (**C**) and PPS (**D**) for patients with BC with high versus low Metadherin expression. *BC* breast cancer, *HR* hazard ratio, *OS* overall survival, *DMFS* distant metastasis-free survival, *RFS* recurrence-free survival, *PPS* progression survival.

Based on the Kaplan–Meier method, a correlation was noted between Metadherin expression and the prognosis of BC. The data in this section was obtained from the TCGA database. In the present study, the median value of Metadherin expression was used as a cut-off score to stratify the patients into high and low groups. High Metadherin expression was associated with significantly worse OS (Fig. 4A), DMFS (Fig. 4B) and RFS (Fig. 4C) compared with low Metadherin expression (OS: P = 0.017; DMFS: P = 0.00063; RFS: P = 3.3 × 10^–08^). Metadherin was not significantly correlated with PPS in breast cancer (Fig. 4D).

### Construction and validation of a nomogram based on independent factors

**Figure 5:**
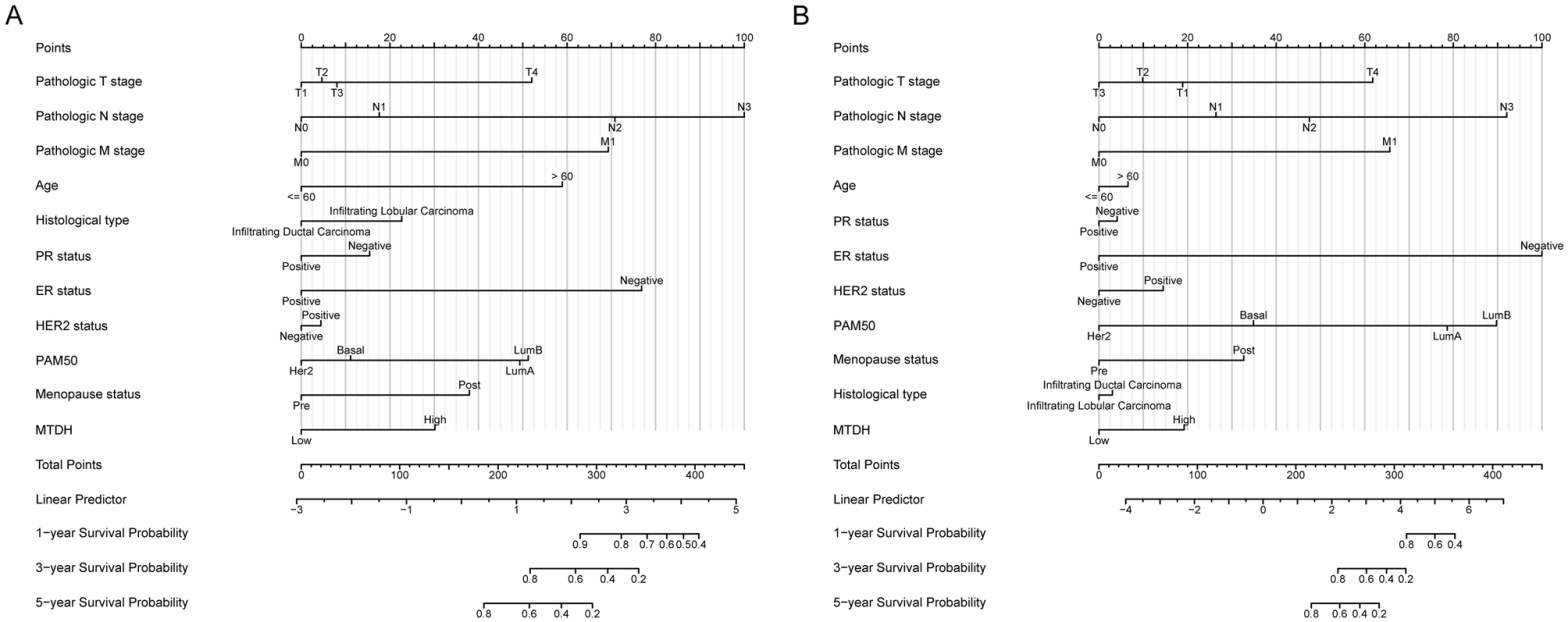
A nomogram and the calibration curves for the prediction of one-, three-, and five-year OS (**A**) and DSS rates (**B**) of patients with BC. *OS* overall survival, *DSS* disease-specific survival, *BC* breast cancer.

Using OS and DSS as independent predictors of prognosis, a nomogram was developed for patients with BC. Nomograms indicated a poor prognosis with a higher total number of points. The points from the nomination chart were assigned to each variable by multivariate Cox analysis. The estimated survival rates of patients with BC at 1, 3, and 5 years were calculated by drawing a vertical line between the total point axis and each prognostic axis, which can aid professionals make clinical decisions for patients with BC (Fig. 5). The bootstrap corrected C-index of the nomogram of OS was 0.825 (95% CI 0.796–0.851), while that of DSS was 0.843 (95% CI  0.799–0.888) indicating that the model had a moderate predictive accuracy for OS and DSS of patients with breast cancer.

### Infiltration of the immune system correlates with the expression of Metadherin

**Figure 6:**
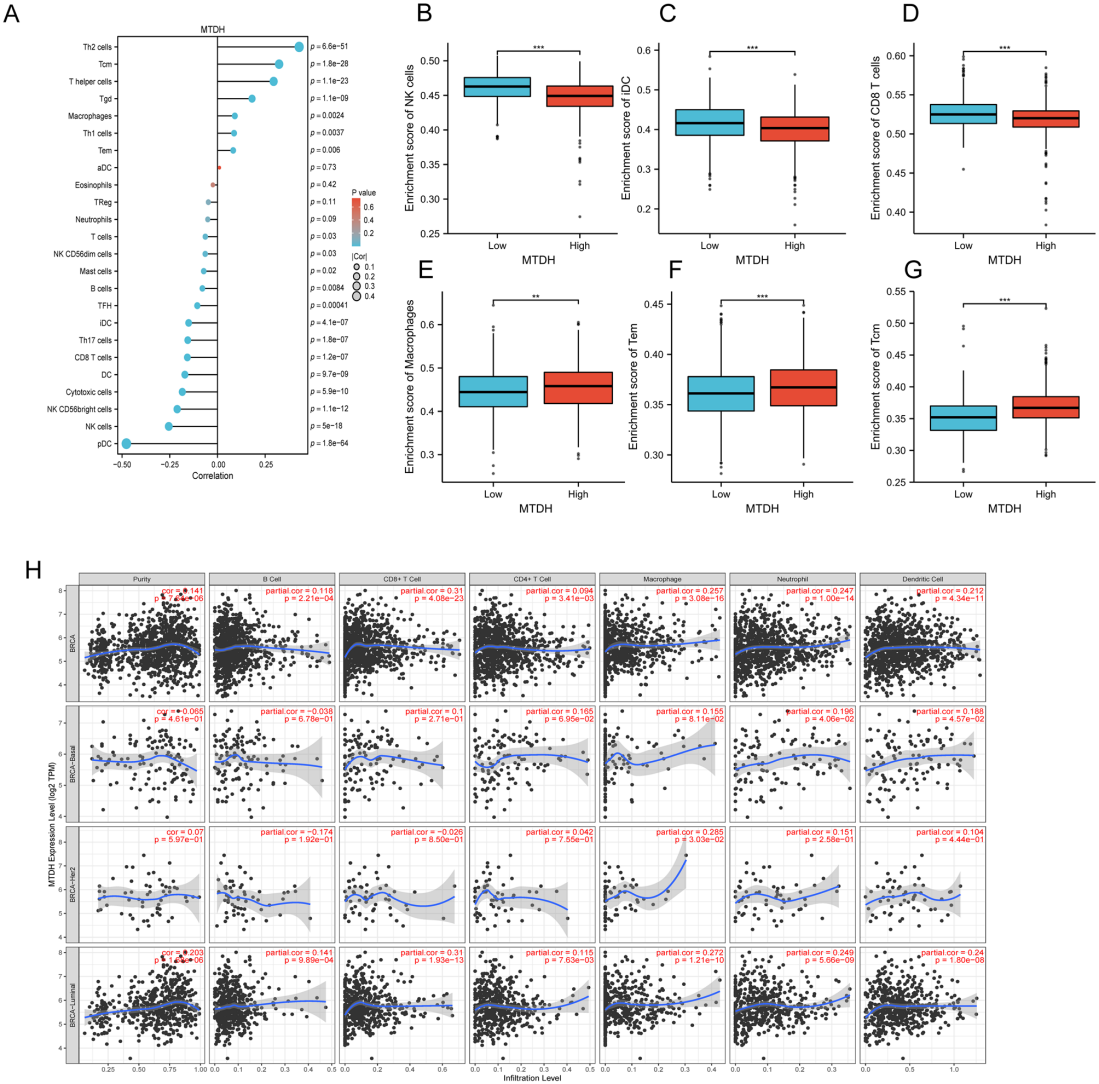
Correlation of Metadherin expression with immune infiltration levels in BC. (**A**) Correlation between Metadherin expression and relative abundance of 24 types of immune cells. The size of the dots corresponds to the absolute Spearman’s correlation coefficient values. (**B**–**G**) Comparison of immune infiltration levels of immune cells (including NK cells, CD8^+^ T cells, iDC cells, macrophages, TEM cells, and Tcm cells) between the high- and low-Metadherin expression groups. (**H**) Correlations between the relative enrichment scores of immune cells and the expression of Metadherin. Unpaired Student’s *t* test was used to analyze the data. The correlation between the expression of Metadherin and these immune cells was investigated using the Spearman’s correlation analysis, and the differences in the level of immune infiltration between the high and low Metadherin expression groups were evaluated using the Wilcoxon rank-sum test. *P < 0.05, **P < 0.01, ***P < 0.005. *BC* breast cancer, *NK cells* natural killer cells, *CD* cluster of differentiation, *iDCs* interstitial dendritic cells, *TEM cells* effector T cells, *pDCs* plasmacytoid dendritic cells.

A significant negative correlation was found between the expression of Metadherin and the infiltration of natural killer (NK) cells (r = – 0.288, P < 0.001), cluster of differentiation (CD)8^+^ T cells (r = – 0.196, P < 0.001), dendritic cells (DCs) (r = − 0.178, P < 0.001), interstitial DCs (iDCs; r = – 0.166, P < 0.001), and T helper (Th) 17 cells (r = – 0.173, P < 0.001; Fig. 6A). Furthermore, NK cells, CD8^+^ T cells, and iDCs were markedly underrepresented in the Metadherin high expression group compared with the Metadherin low expression group (all P < 0.001; Fig. 6B–D). A significant difference was noted between the enrichment scores of macrophages, Tcm, Metadherin high expression, and Metadherin low expression groups (all P < 0.005; Fig. 6E–G). Figure 6H showed the frequency distribution of immune cell infiltrating tumors with different Metadherin mutation status in different subgroup in BC.

### Identification of differentially expressed genes (DEGs) in BC and protein–protein interaction (PPI) network analysis

**Figure 7:**
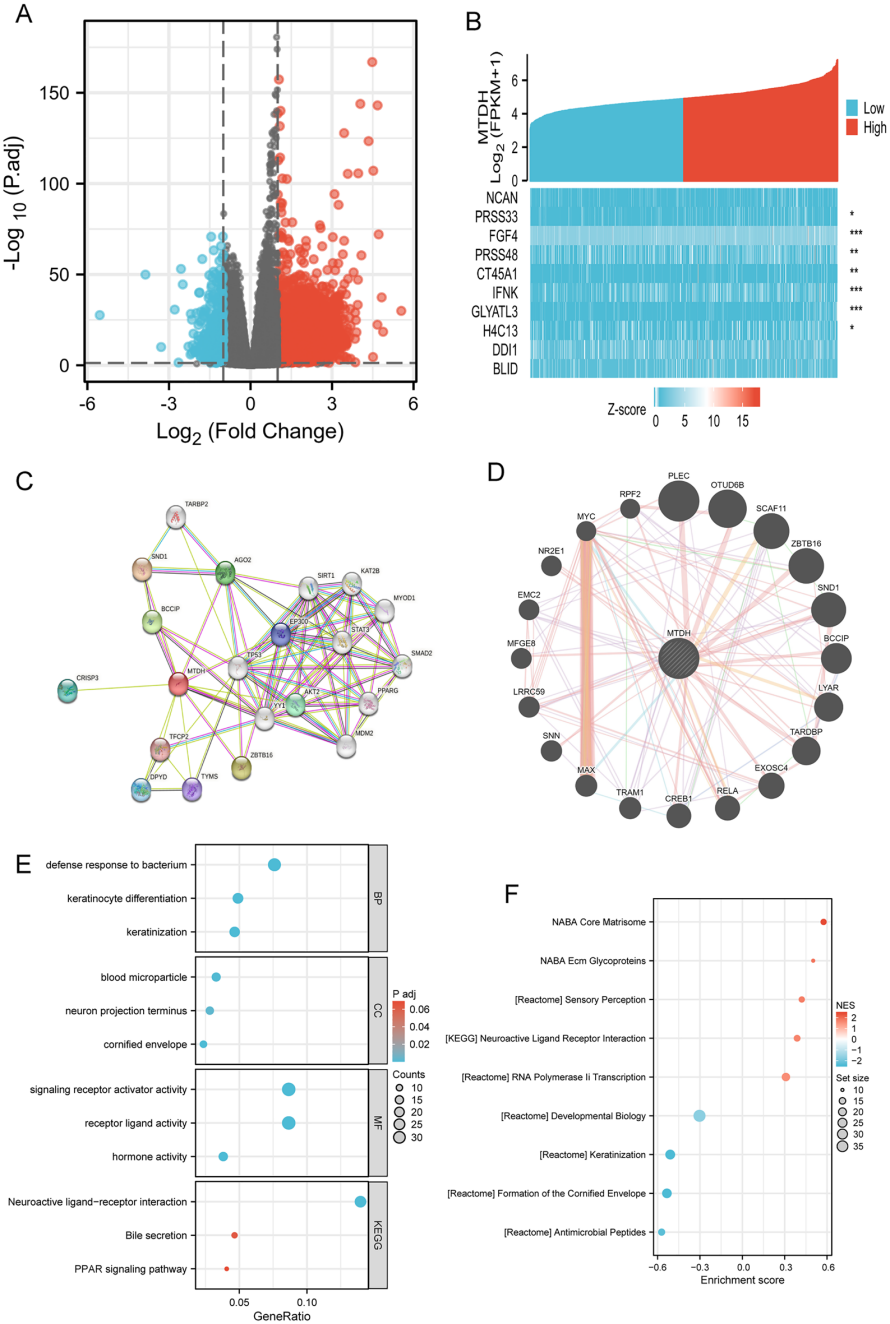
Metadherin-related DEGs and functional enrichment analysis of Metadherin in BC using GO and KEGG. (**A**) Volcano plot of DEGs. Blue and red dots indicate the significantly downregulated and upregulated DEGs, respectively. (**B**) Heatmap of the correlation between Metadherin expression and the top 10 DEGs. (**C**) STRING database PPI map. (**D**) GeneMANIA database analysis indicates that Metadherin interacts with proteins. (**E**) GO and KEGG analysis of DEGs. (**F**) GSEA analysis of DEGs. The correlation between the expression of the top 10 DEGs and Metadherin was evaluated using Spearman’s correlation analysis. *DEGs* differentially expressed genes, *BC* breast cancer, *GO* gene ontology, *KEGG* Kyoto encyclopedia of genes and genomes, *STRING* search tool for the retrieval of interacting genes/proteins, *PPI* protein–protein interaction. *P < 0.05, **P < 0.01, and ***P < 0.001.

A total of 1279 genes were differentially expressed between the groups with high and low expression levels of Metadherin, including 200 upregulated DEGs (15.6%) and 1079 downregulated DEGs (84.4%; adjusted P value < 0.05, |Log2-FC|> 2; Fig. 7A). Subsequently, the relationship between the top 10 DEGs (including NCAN, PRSS33, FGF4, PRSS48, CT45A1, IFNK, GLYATL3, H4C13, DDI1, and BLID) and METADHERIN was assessed (Fig. 7B). The STRING online tool was used to establish a PPI network, and subsequently the hub genes were determined. As shown in Fig. 7C, the network of the DEGs was complex, and the top 10 hub genes were the following: DPYD, TFCP2, TYMS, CRISP3, AKT2, BCCIF, SND1, AGO2, EP300, and ZBTB16. Based on Genemania, the gene interaction networks for the neighboring genes of Metadherin, including PLEC, were depicted (Fig. 7D). The following neighboring genes were identified: OTID4B, SCAF11, ZBTB16, SND1, BCCIP, LYAR, TARDBP, EXOSC4, and RELA.

### Functional enrichment analysis includes gene ontology (GO), Kyoto encyclopedia of genes and genomes (KEGG) analyses and gene set enrichment (GSEA) analyses

According to the GO enrichment analysis, DEGs were enriched in GO terms, such as ‘defense response to bacterium’, ‘keratinocyte differentiation’, ‘blood microparticle’, ‘signaling receptor activator activity’ and ‘receptor ligand activity’ (Fig. 7E). A KEGG pathway analysis also revealed significant DEG-enriched pathways, including ‘neuroactive ligand-receptor interaction’, ‘Bile secretion’ and ‘PPAR signaling pathway (Fig. 7E). GSEA analysis revealed significant DEG-enriched pathways, including ‘NABA Core Matrisome’, ‘NABA Ecm Glycoproteins’, ‘developmental biology’, ‘keratinization’, and ‘Formation of the Cornified Envelop’ (Fig. 7F).

### BC cell proliferation and migration are influenced by Metadherin expression

**Figure 8:**
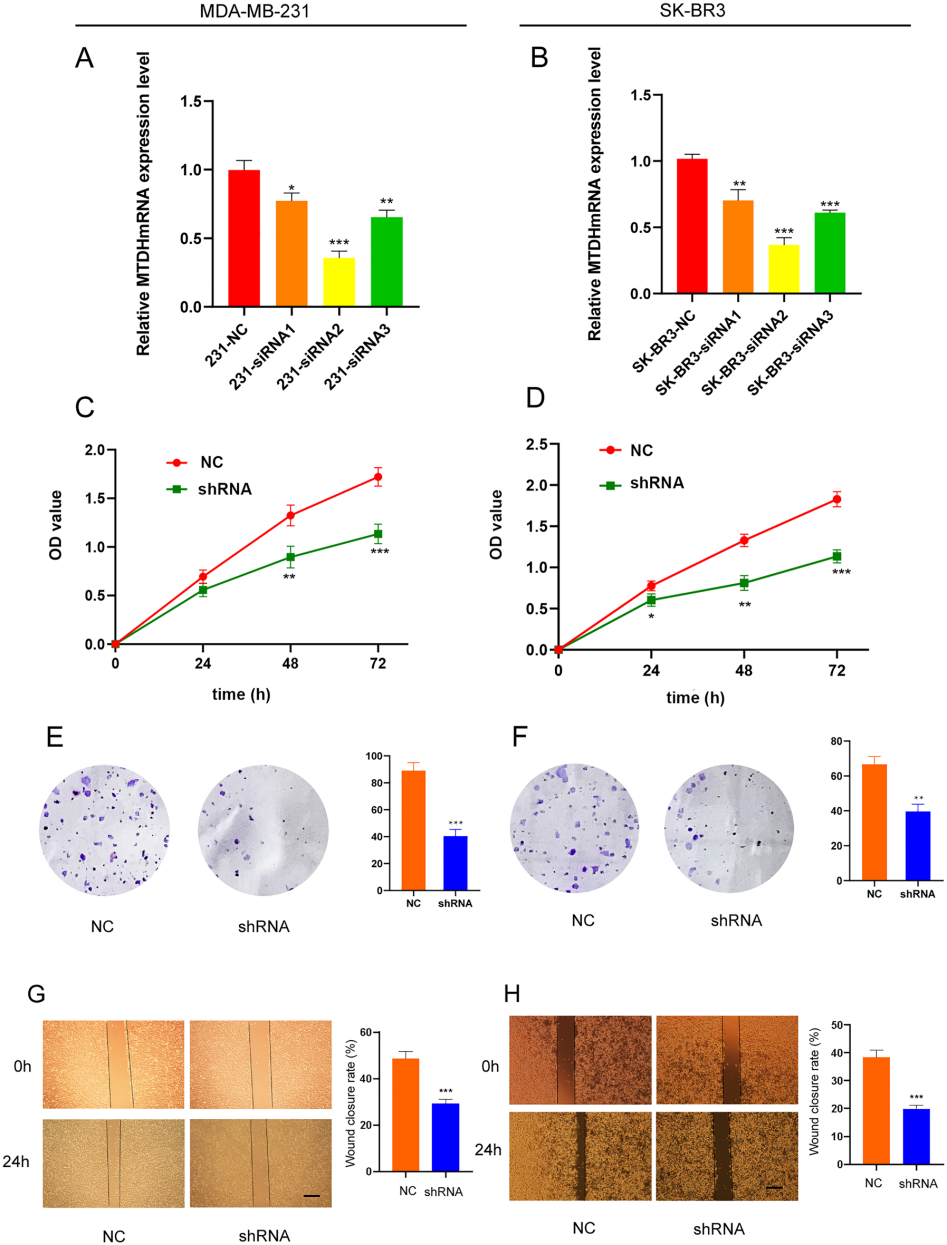
Downregulation of Metadherin inhibits cell proliferation and migration of breast cancer cells. RT-qPCR analysis of Metadherin expression levels in MDA-MB-231 (**A**) and SK-BR3 (**B**) cells transfected with Metadherin-shRNAs and NC. Cell proliferation in MDA-MB-231 (**C**) and SK-BR3 (**D**) cells transfected with Metadherin -shRNA and NC. Colony formation assays for MDA-MB-231 (**E**) and SK-BR3 (**F**) cells transfected with Metadherin-shRNA and NC. Wound healing assays for MDA-MB-231 (**G**) and SK-BR3 (**H**) cells transfected with Metadherin -shRNA and NC. Dunnett’s post-hoc test was used to compare the levels of Metadherin expression between BC and normal breast epithelial cells. Mann–Whitney *U*-test or one-way ANOVA and unpaired Student’s t‑test was used to analyze the data. *RT-qPCR* reverse transcription-quantitative PCR, *NC* negative control, *shRNA* short hairpin RNA, *OD* optical density.

Transfection experiments as CCK-8 assays, colony assays and colony formation assays were performed in vitro to further understand the biological role of Metadherin in BC. In both MDA-MB-231 and SK-BR3 cells, Metadherin was highly expressed. In order to determine which shRNA plasmid exhibited the greatest effect, MDA-MB-231 and SK-BR3 cells were transfected with three shRNA plasmids, and RT-qPCR results revealed shRNA2 to be the most effective (Fig. 8A and B). In order to investigate the effects of Metadherin on BC proliferation and migration, CCK-8 assays, and colony formation assays were performed along with wound healing tests. The proliferative (Fig. 8C and D), colony formation (Fig. 8E and F) and migratory (Fig. 8G and H) properties of BC cells were significantly suppressed following transfection by shMetadherin.

## Discussion

The correlation between Metadherin expression and BC and immune infiltration is based on data from public databases. Similarly in the histological assay, a significant increase has been noted in the mRNA expression of Metadherin in invasive BRACs compared with the corresponding expression in adjacent tissues, while increasing Metadherin mRNA levels are related to poor prognosis in patients with BC.

Oncoprotein Metadherin is widely involved in the malignant behavior of various tumors, and it has been shown to affect tumor cell proliferation, apoptosis, invasion, metastasis, and angiogenesis^15–19^. Several signaling pathways have been shown to be activated by Metadherin in tumors, including NF-κB, PI3K/AKT, Wnt/b-catenin, and MAPK^19,20^. According to Emdad et al.^21^ BC cells express high levels of Metadherin, which may promote proliferation and invasion. Metadherin expression is associated with proliferative and metastasizing properties in triple-negative BC, according to Liu et al.^22^, while Tokunaga et al.^7^ indicated that Metadherin was expressed at low levels in patients with BC and high ER and/or PR expression.

In the current study, public data were analyzed and the results indicated that high Metadherin expression could predict poor prognosis in BC, including OS and DSS. In addition, a ROC curve was plotted and a nomogram was established to observe the efficacy of Metadherin as a prognostic biomarker. The results indicated that the Metadherin gene was a variable independent of other clinical factors that could guide the prognosis of BC. Therefore, a series of related functional assays, such as cell proliferation assays, wound healing assays and colony formation assays, were performed in BC cells and the data indicated that knockdown of Metadherin expression inhibited their proliferation and migration, further suggesting a possible role for Metadherin as an oncogene.

Gene expression is generally silenced by DNA methylation, a common epigenetic mechanism^23^. Further investigation of the underlying mechanism of Metadherin overexpression in BC indicated that its DNA hypomethylation may be associated with Metadherin overexpression. Aberrant DNA methylation is an epigenetic feature of tumors, leading to tumor development and progression by silencing oncogenes and activating oncogenes, so we performed corresponding analysis of the methylation level of Metadherin in breast cancer tissues, expecting its suggestive role in promoting breast carcinogenesis and development. It has been shown that patients with BC and hypomethylated Metadherin exhibit a poor prognosis. It is difficult to determine the DNA methylation level of Metadherin in solid tumors, and research in the field of BC has been contradictory.

Pathway enrichment analysis of Metadherin revealed a possible association with keratinocyte differentiation, blood microparticle, NABA core matrisome and NABA Ecm glycoproteins, which may also suggest a possible mechanism for the high expression of Metadherin in BC tissues and its subsequent association with poor disease prognosis; however, this requires confirmation by more in-depth studies.

An association between Metadherin expression and the tumor microenvironment was initially established by bioinformatic analysis in the present study. Infiltrating immune cells are major factors affecting the outcome of immunotherapy and the prognosis of patients^24,25^. Several studies have shown that tumor-infiltrating lymphocytes have an impact on the clinical outcomes of immunotherapy in patients with melanoma, colorectal cancer, and ovarian cancer^26,27^.Tumor microenvironments including the stromal influences and macrophages are important in the initiation and progression of BC^28,29^. The prognostic value of tumorinfiltrating immune cells has been demonstrated in solid malignant tumors, which are affected by the type, density, and location of immune cells^30^. Additionally, the prediction of response to neoadjuvant chemotherapy and immune checkpoint inhibition (ICI) treatment has also been demonstrated by infiltrating immune cells^31,32^. Therefore, evaluating the presence of infiltrating immune cells in breast cancer not only has the potential to enhance the effectiveness of ICI treatment but also holds promise as a predictive marker for ICI therapy. Considering that the group with high Metadherin expression displayed an enrichment in pathways related to leukocyte migration and regulation of immune response signaling, we proceeded to assess the correlation between Metadherin expression and the levels of infiltrating immune cells. The analysis revealed Metadherin correlated with macrophages, pDC, NK cells, and CD8 + T cells. Both activated pDCs and NK cells, known as innate immune cells, have demonstrated the ability to halt the growth of breast cancer cells^24^. Improved prognosis in patients with breast cancer has been linked to the existence of CD8 + T cells^24^. Furthermore, clinical samples of patients with BC were analyzed in order to confirm that Metadherin is a prognostic marker of this disease. These findings suggest that the progression and prognosis of breast cancer could be influenced by the overexpression of Metadherin, which regulates the levels of infiltrating immune cells. Because Metadherin expression is elevated in breast cancer tissues and is strongly correlated with immune micro-relationships, the detection of Metadherin in breast cancer tissues may be useful for prognostic speculation or for decision-making regarding immunotherapy.

In this novel research, bioinformatics has revealed the correlation between Metadherin and the tumor microenvironment (TME) in breast cancer for the very first time. Additionally, through the analysis of a series of clinical samples from breast cancer patients, this investigation has substantiated Metadherin’s potential as a prognostic indicator for breast cancer. It is worth noting that our conclusions are contingent upon data obtained from publicly accessible databases, thus containing certain constraints. It is imperative to acknowledge that the association between Metadherin and prognosis might vary in accordance with the updates in databases. Moreover, the relationship between Metadherin mRNA levels and various immune cell categories, as well as markers, may also exhibit alterations based on sequencing data derived from public databases. Conversely, as more resources accumulate, the stratification of data will become more refined, thereby enhancing the reliability of the results. Nonetheless, additional experimentation, larger sample sizes, longer follow-ups are indispensable to validate the outcomes determined in this study.

## Conclusion

The present scientific investigation has examined the relevance of Metadherin and its significance for predicting outcomes in breast cancer. In broad terms, Metadherin is found to be elevated in tissues affected by breast cancer. Additionally, there exists a correlation between Metadherin, immune infiltration, and survival rates in breast cancer. Taken together, these findings indicate the necessity for further exploration of Metadherin in breast cancer and its potential as a biomarker for prognosticating the outcomes of breast cancer patients.

## Materials and methods

### Bioinformatic analysis of RNA-sequencing (seq) data

The cancer genome atlas (TCGA)-breast adenocacinoma (BRCA) (http://tcga.xenahubs.net) dataset was used to normalize the RNA-seq data and to identify clinical features associated with BRCA mutations. For subsequent analysis, 1087 BCs and 113 whole breast tissues were downloaded, with three levels of HTSeq fragments.

### Collection of pathological samples

From August 2020 to April 2023, a total of 40 paraffin-embedded BC tissues and their corresponding normal tissue samples were collected from the Breast Center of the Fourth Hospital of Hebei Medical University and the Breast Surgery Department of Xingtai People’s Hospital. The present study was approved by the Medical Ethics Committee of the Fourth Hospital of Hebei Medical University and Xingtai People’s Hospital, and was carried out in accordance with the Declaration of Helsinki.

### Database analysis of the tumor immune estimation resource

This method allows for the analysis of gene expression and tumor-infiltrating immune cells across a wide range of cancer types using a centralized database (TIMER 2.0, http://cistrome.shinyapps.io/timer/). In a previous study using TIMER, the expression levels of Metadherin were examined in tumors and were compared with those noted in the normal tissues. TIMER was utilized to investigate the association of Metadherin with the expression levels of immune cell infiltrating tumors and immune cell markers. The association between immune cell infiltration and gene expression in TIMER was evaluated using Spearman’s correlation analysis.

### A portal for data analysis for University of Alabama at Birmingham Cancer (UALCAN)

UALCAN data analysis portal (http://ualcan.path.uab.edu/index.html) allows online analysis of differential gene expression between cancer types and their corresponding normal tissues using TCGA‐RNA seq datasets and clinical datasets^33^. With the exception of providing survival prognostic data, this website examines 31 cancer types based on gene expression differences.

### Nomogram construction

The "rms" and "survival" packages in R were used to construct nomogram models based on Metadherin expression. The calibration curves were used to estimate the consistency between actual and predicted survival.

### Differentially expressed gene analysis

According to the median score of Metadherin expression, patients with breast cancer in TCGA were divided into high and low Metadherin expression groups. The R package DESeq2 was used to perform the differentially expressed gene (DEG) analysis between these two groups, and adjusted p value < 0.05, and |log2-fold-change (FC)|> 2 were set as the thresholds of DEGs.

### Functional enrichment analysis

Functional enrichment analyses, including Gene Ontology (GO) and Kyoto Encyclopedia of Genes and Genomes (KEGG) analysis^34–36^, were implemented for the DEGs using the R package GOplot (version 1.0.2).

### Analyses of immune infiltration

In the present study, the levels of tumor immune infiltration were estimated via the single sample gene set enrichment analysis (GSEA) method with the ‘GSVA’ R package using the datasets from the TCGA and BRCA trials. The correlation of Metadherin expression and immune cell type infiltration was investigated using Spearman’s rank correlation coefficient analysis. The ‘ggplot2’ R package was used to generate graphs and figures, and Spearman and Wilcoxon rank sum tests were used to determine the effects of their deviations.

### Cultures and cell lines

The human BC cell lines MCF‐7, SKBR‐3, and MDA‐MB‐231 and the normal breast epithelial cell line MCF10A were obtained from Procell Life Science & Technology Co., Ltd. SKBR‐3, MCF‐7, and MDA‐MB‐231 cells were routinely cultured in DMEM (Gibco; Thermo Fisher Scientific, Inc.) supplemented with 10% (v/v) fetal bovine serum (Gibco; Thermo Fisher Scientific, Inc.) and a 1% (v/v) penicillin and streptomycin (Beyotime Institute of Biotechnology). MCF10A cells were cultured in mammary epithelial cell medium (Procell Life Science & Technology Co., Ltd.) supplemented with 10% horse serum, EGF, hydrocortisone, insulin, and 1% penicillin‐streptomycin. Standard cell culture methods were used to passage all cell lines in an incubator at 37 °C with 5% CO_2_.

### Reverse transcription-quantitative PCR (RT‐qPCR)

A RNAiso Plus Kit cat (no. 9109; Takara Biotechnology Co., Ltd.) was used to isolate total RNA from SK-BR3, MCF-7, MDA-MB-231, and MCF10A cells according to the manufacturer’s protocol. Reverse transcription was conducted with the PrimeScriptTM RT Reagent Kit and the genomic DNA Eraser; a total of 1000 ng mRNA were converted to cDNA (cat. no. RR047; Takara Bio, Inc.) according to the manufacturer’s protocol. A LightCycler® 96 instrument (Roche Diagnostics) was used to perform qPCR analysis using TB Green Premix Ex Taq (cat. no. RR420; Takara Bio, Inc.) to detect the expression levels of the target genes. The following primer pairs were used for qPCR: Metadherin forward, 5′‐AAATGGGCGGACTGTTGAAGT‐3′ and reverse, 5′‐CTGTTTTGCACTGCTTTAGCAT‐3′; and GAPDH forward, 5′‐CATTGACCTCA A CTACATGGTTT‐3′ and reverse, 5′‐GAAGATGGTGATGGG ATTTCC‐3′. The thermal cycling conditions for qPCR were the following: 95 °C for 5 min, followed by 40 cycles of 95 °C for 10 s and 60 °C for 30 s. The relative cycle threshold of the endogenous control gene GAPDH was used to normalize the rate of relative mRNA expression levels based on the relative cycle threshold using the standard 2^–ΔΔCq^ method^37^. Triplicates of each experiment were performed.

### Cell transfection

Transfections with Metadherin-short hairpin (sh) RNAs and negative controls were performed using Lipofectamine® 3000 (Invitrogen; Thermo Fisher Scientific, Inc.) at a concentration of 100 nM in SK-BR3 and MDA-MB-231 cells at 37˚C for 6 h. The following 3 specific Metadherin short hairpin RNAs (shRNAs): sh Metadherin -1, 5′AGGAATAAAGGATTCTGAT3′; sh Metadherin -2, 5′-AAGTCAAATACCAAGCAAA-3′; and shMetadherin-3, 5′-AACTTACAACCGCATCATT-3′, as well as a negative control shRNA (shNC), 5′-TTCTCCGAACGTGTCACGT-3′. Transfection was followed by 48-h experiments.

### Cell proliferation assay

The Cell Counting Kit-8 (CCK-8; Dojindo Laboratories, Inc.) assay was used to measure cell proliferation. SK-BR3 or MDA-MB-231 cells were plated at a density of 2 × 10^3^ cells per well in 96-well plates. Each well was subsequently incubated at 37 °C for 4 h following an initial incubation for 24, 48, and 72 h at 37 °C. Microplate readers (Multiskan Sky; Thermo Fisher Scientific, Inc.) were used to determine the absorbance of each well at 450 nm.

### Wound healing assay

A total of 2 × 10^5^ SK-BR3 or MDA-MB-231 cells were incubated at 37 °C in a 35 mm^2^ petri dish at 48 h following transfection. Prior to the removal of detached cells, a 200-µl pipette tip was used to scratch the monolayer. The cells from the remaining wound were cultured in serum-free culture media and wound closure was observed. Using an Olympus (Olympus Corporation) light microscope (magnification × 100), the images of the wounds were obtained at 0 and 24 h following scratching. The wound closure was calculated using ImageJ software version 1.47. The mobility ratio was used as a measure of the migratory ability and was calculated as follows: Mobility ratio = (scratch width at 24 h-scratch width at 0 h)/scratch width at 0 h.

### Colony formation assay

Colony formation assays were performed to detect cell proliferation. Transfected MBA-MD-231 and SK‑BR3 cells were seeded at 1 × 10^3^ cells/well in six‑well plates and incubated with 5% CO_2_ at 37 °C. After 15 days, colonies were visible to the naked eye. These colonies were fixed with 4% paraformaldehyde for 15 min at ambient temperature and stained with 1% crystal violet (Beyotime Institute of Biotechnology) for 15 min at ambient temperature. The number of colonies (containing > 50 cells) was counted microscopically.

### Statistical analysis

A bioinformatic analysis was carried out using R version 4.2.2 (https://cloud.r-project.org/). Comparisons between or among groups were performed using unpaired Student’s *t* test, paired Student’s *t* test, Mann–Whitney *U*-test or one-way ANOVA. Tukey’s HSD was used as a post hoc test following ANOVA. To determine the predictive value of Metadherin expression for BC diagnosis, receiver operating characteristic (ROC) analysis was conducted between BC tumors and paracancerous tissues. In order to determine the relationship between Metadherin expression levels and patient survival, Kaplan–Meier (KM) plotter was used. The TCGA-BRCA dataset was divided into high and low Metadherin expression groups based on median expression. An R package called 'Forest plot' was used to visualize the results of a one-way Cox survival analysis. The statistical analysis of clinical features and Metadherin expression was conducted using the Mann U Whitney, Wilcoxon signed rank, and Fisher’s exact tests, as well as logistic regression. Spearman rank correlation test was used for the correlation analysis between Metadherin and co-expressed genes. Dunnett’s post-hoc test was used to compare the levels of Metadherin expression between BC and normal breast epithelial cells. Each experiment was repeated three times, and the data are presented as mean ± standard deviation. P < 0.05 was considered to indicate a statistically significant difference.

### Ethics approval and consent to participate

All procedures performed in the present study involving human participants were in accordance with The Declaration of Helsinki (as revised in 2013). The study was approved by the Institutional Ethics committee of The Fourth Hospital of Hebei Medical University (approval no. 2021KY056) and Xingtai People’s Hospital (approval no. 2021[030]). Written informed consent was obtained from each patient.

## Data Availability

The datasets generated and/or analyzed during the current study are not publicly available due to restrictions applied by Xingtai People’s Hospital but are available from the corresponding author on reasonable request.
